# Tissue-Specific Natural Synthesis of Galanthaminein *Zephyranthes* Species and Its Accumulation in Different In Vitro-Grown Organs Following Methyl Jasmonate Treatment

**DOI:** 10.3390/plants13141931

**Published:** 2024-07-13

**Authors:** Rukaya Syeed, A. Mujib, Yashika Bansal, Mohammad Mohsin, Afeefa Nafees, Moien Qadir Malik, Jyoti Mamgain, Bushra Ejaz, Yaser Hassan Dewir, Katalin Magyar-Tábori

**Affiliations:** 1 Cellular Differentiation and Molecular Genetics Section, Department of Botany, Jamia Hamdard, New Delhi 110062, India; rukayasyeed@gmail.com (R.S.); yashikab333@gmail.com (Y.B.); mohammadmohsin_sch@jamiahamdard.ac.in (M.M.); afeefanafees9045@gmail.com (A.N.); malikmoien199@gmail.com (M.Q.M.); jyotimamgain93@gmail.com (J.M.); bushra.libra91@gmail.com (B.E.); 2 Plant Production Department, College of Food and Agriculture Sciences, King Saud University, Riyadh 11451, Saudi Arabia; ydewir@ksu.edu.sa; 3 Research Institute of Nyíregyháza, Institutes for Agricultural Research and Educational Farm (IAREF), University of Debrecen, P.O. Box 12, 4400 Nyíregyháza, Hungary; mtaborik@agr.unideb.hu

**Keywords:** Amaryllidaceae alkaloids, bioactive compounds, elicitor, elicitation, GC-MS, HPTLC

## Abstract

Galanthamine is an immensely valuable alkaloid exhibiting anti-cancer and antiviral activity. The cultivation of plant tissues in in vitro conditions is a good source for the synthesis and enrichment of secondary metabolites of commercial interest. In this study, the Amaryllidaceae alkaloid galanthamine was quantified in three *Zephyranthes* species, such as *Zephyranthes candida*, *Zephyranthes grandiflora*, and *Zephyranthes citrina*, and the impact of the methyl jasmonate (MJ) signaling molecule on galanthamine accumulation was monitored in in vitro-derived plant tissues. This is the first ever study of the MJ-regulated accumulation of galanthamine in in vitro-grown *Zephyranthes* tissues. Shoot regeneration was obtained in all three *Zephyranthes* species on Murashige and Skoog (MS) medium containing 2.0 mgL^−1^ benzylaminopurine (BAP) + 0.5 mgL^−1^ naphthalene acetic acid (NAA). The regenerated shoots were rooted on a medium containing 2.0 mgL^−1^ indole butyric acid (IBA). A GC-MS study of *Zephyranthes* extracts revealed the presence of 34 phyto-compounds of varied levels with therapeutic activities against diseases. The galanthamine content was quantified in plant parts of the three *Zephyranthes* species using high-performance thin layer chromatography (HPTLC); the maximum was found in *Z. candida* bulb (2.41 µg g^−1^ dry wt.), followed by *Z. grandiflora* (2.13 µg g^−1^ dry wt.), and then *Z. citrina* (2.02 µg g^−1^ dry wt.). The galanthamine content showed bulb > leaf > root source order. The in vitro-generated plantlets were treated with different MJ concentrations, and the galanthamine yield was measured in bulb, leaf, and root tissues. The highest galanthamine content was recorded in bulbs of *Z. candida* (3.97 µg g^−1^ dry wt.) treated with 150 µM MJ, showing an increase of 64.73% compared to the control. This accumulation may be attributed to MJ-induced stress, highlighting the potential commercial synthesis of galanthamine in vitro.

## 1. Introduction

The *Zephyranthes* is a bulbous plant of the family Amaryllidaceae. There are some 70 species; these *Zephyranthes* species are distributed to several regions like Cuba, Puerto Rico, Guadeloupe, Martinique and Mexico [1]; and it was also found in North, Central, South America, Asia, Australia, and some other parts of the world [2]. The genus *Zephyranthes* is a temperate and tropical plant that grows well in rich, moist, drained soil with an acidic to neutral pH. The parts of *Zephyranthes*, like bulbs and leaves, are used in traditional medicine. The *Z. candida* species was exploited against tumors in Peru. In China, *Z. rosea* was used against breast cancer. Similarly, *Z. candida* leaves were used to treat diabetes in Africa. Besides, the plant parts are used to treat common ailments like headaches, cough, colds, and complex disorders like breast cancer, tuberculosis, rheumatism, and other forms of tumors [3].

Alkaloids, being a diverse class of secondary metabolites, are present in about 20% of the species in the plant kingdom, and about 20,000 alkaloids have been purified and isolated from various plant species [4]. A class of characteristic compounds of Amaryllidaceae (called Amaryllidaceae alkaloids) is among the top 20 medicinal in the whole plant kingdom, and so far, about 600 Amaryllidaceae alkaloids have been isolated [5]. The prime alkaloids present in this class are lycorine, homolycorine, haemanthamine, tazettine, crinine, pancratistatin, and galanthamine [6]. Amaryllidaceous alkaloids, along with other structurally diverse alkaloid groups like phenylpropanoid and isoquinoline, demonstrate several important pharmacological activities [7]. The bulb and leaf extracts of *Zephyranthes* are conventionally utilized in Chinese medicine due to their anti-inflammatory, anti-bacterial, antiviral, anticarcinogenic, and antidiabetic properties. In the genus *Zephyranthes*, phytochemical investigation began in the 19th century, and many compounds were identified (alkaloids, flavonoids, phospholipids, terpenes, and ceramides), among which the alkaloids are very valuable [8]. In *Crinum*, a related genus of *Zephyranthes*, the bulbs have also been extensively studied and used to treat wounds, mental illness, and tumors [9].

**Figure 1:**
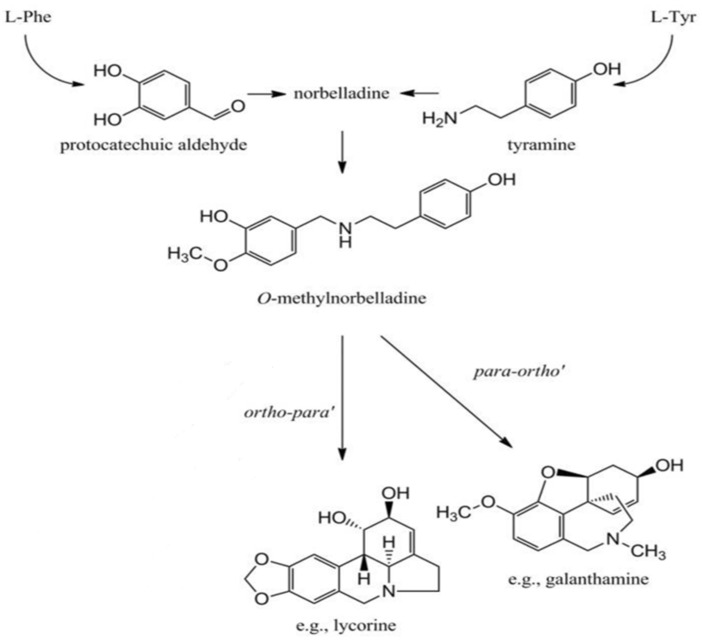
Biosynthesis of galanthamine and lycorine [10].

The biosynthesis of Amaryllidaceae alkaloid galanthamine and related lycorine starts with the condensation of amino acids involving bond formation and functional group addition in forming structurally diverse compounds. The condensation produces precursors, and the common precursor called Norbelladine is synthesized in all Amaryllidaceae alkaloids [10]. Likewise, the Shikimate biosynthetic pathway condensates L-phenylalanine and L-tyrosine to form primary precursor. Similarly, the phenylpropanoid and other core pathways form 3,4-dihydroxybenzaldehyde (3,4-DHBA), which produces the precursor norbelladine. In other instances, the methylation of norbelladine and phenol coupling synthesizes different alkaloids [11]. The ortho-para coupling forms lycorine, and the para-ortho coupling produces galanthamine. The biosynthesis of galanthamine and lycorine is represented in Figure 1.

Galanthamine, the compound of interest, is an isoquinoline alkaloid found in many Amaryllidaceae species. It was discovered in 1950 in the bulb and lower parts of Caucasian snowdrops (*Galanthus woronowi*). Galanthamine is potent in curing several disorders like dementia, facial nerve paralysis, Schizophrenia, and Alzheimer’s disease [12,13]. This isoquinoline galanthamine is used against Alzheimer’s disease, under the Food and Drug Administration in the United States, bearing the commercial name Reminyl [14]. The Amaryllidaceae alkaloids are present in less than 1% (dry weight basis) of plants, and 50% of the commercial products are obtained directly or indirectly from plants; thus, in vitro cell culture techniques may be an alternative way to improve secondary metabolite yield [15]. Moreover, the bioactive compounds are often restricted to certain genera, species, and varieties, and accumulated in specific organs different from the chemical synthesis site [16]. Due to low and organ-specific accumulation, variable ecological and phytogeographical distribution, and difficult extraction procedures, plant tissue culture techniques have been employed to detect and enhance the production of secondary metabolites using callus [17], suspension [18], hairy root [19], and shoot culture [20]. The metabolomic study through gas chromatography-mass spectrometric (GC-MS) has recently picked up momentum in frontier areas of research, including the pharma sector. It identifies and quantifies compounds in a mixed population of biological preparations [21]. In recent times, metabolite profiling via GC-MS has been performed on several important plants [22,23], but unfortunately, not enough information is available on *Zephyranthes* grown in vivo or in vitro. In some cases, secondary metabolite accumulation is impacted by stress, which facilitates important bioactive compound synthesis. Various plant biotechnological methods, like precursor feeding, medium, and plant growth regulator’s use, immobilization, and elicitation, have been employed in stress-mediated enriched secondary metabolite synthesis [24]. Elicitation is an important technique widely used in increasing the synthesis of secondary metabolites [25]. The elicitor is added in trace amounts, induces stress in cultured tissues, changes the physiological and biochemical dynamics of cells, mediates gene expression, and activates the production of phytocompounds [26,27].

Methyl Jasmonate (MJ) is a specific signaling molecule induced by wounding or pathogen invasion and transported both locally and systemically in plants [28]. Jasmonic acid is a stress hormone produced endogenously to regulate plant defense mechanisms and stress against various biotic and abiotic challenges [29]. MJ is a methyl ester of jasmonic acid and a most common elicitor used in the plant kingdom (from gymnosperms to angiosperms), reporting almost 60% of the elicitation process of alkaloids, phenylpropanoids, and phenols [30]. The addition of exogenous stress triggers various signal transduction pathways, stimulates a variety of genes, and synthesizes a number of proteins, which protect cells from damage caused by reactive oxygen species (ROS) [31]. Being low molecular weight, MJ is easily transported, acts as a secondary messenger in many pathways, and activates disease-resistant genes in synthesizing PR proteins for systemic acquired resistance [32]. It is the only signaling element that directly regulates defense-related genes in the production of all kinds of secondary metabolites, such as alkaloids, phenols, terpenes, flavonoids, and phenylpropanoids [33]. Optimization of MJ concentration, growth stage, and exposure time are critical for enhancing secondary metabolite accumulation [34]. MJ has been widely used for enhancing secondary metabolites like podophyllotoxin in *Linum album* [35], catharanthine from *Catharanthes roreus* [36], centellosides from *Centella asiatica* [37], ginsenosides from *Panax ginsing* [38]. Previous studies confirmed a few-fold increases in secondary metabolite yield on addition of exogenous MJ. The hairy root culture of *Linum tauricum* increased podophyllotoxin yields up to 1.2 fold [39]. Recent studies revealed enhanced secondary metabolite yield up to several folds in in vitro shoot cultures of *Solenostemon scutellarioide*, callus cultures of *Rosa hybrida* [40], and cell suspension cultures of *Catharanthus roseus* [41]. In addition, MJ has been extensively studied for the elicitation of triterpenoids, saponins, and phytosterols [31]. In the current study, the alkaloid galanthamine was quantified in naturally grown and tissue culture plant organs like the bulb, leaf, and root of *Zephyranthes* species, namely *Z. candida*, *Z. grandiflora*, and *Z. citrina.* In addition, the influence of MJ elicitor on the synthesis and accumulation of galanthamine was assessed.

## 2. Results

### 2.1. GC-MS Study and Bioactive Compounds

**Figure 2:**
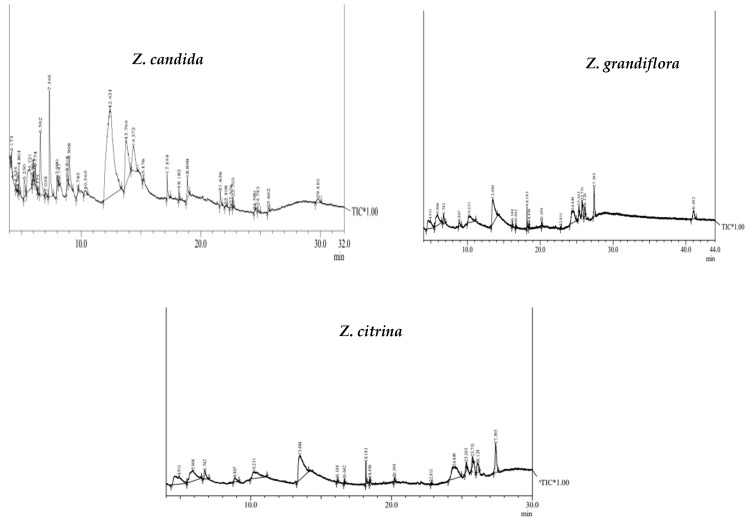
GC-MS spectra of *Zephyranthes* bulb extracts showing Amaryllidaceae alkaloids peaks.

In the present study, the methanolic extracts of bulb parts from three species were used for GC-MS analysis. The chromatographic study revealed the presence of 34 phyto-compounds at varied levels, exhibiting various phytochemical activities. The GC-MS spectra of three bulb species are presented in Figure 2, while the common chemical constituents with retention time (RT) area % are presented in Table 1. The common bioactive compounds present in methanol fractions of *Zephyranthes* species include guanosine, alpha D-galactopyranoside, 9-octadecenoic acid, Prectazettine alpha, D-galactopyranoside, 1,23 propanetriol, diacetate, cyclopropylmethanol, 5-hydroxymethylfurfyryl, 2-hydroxy-gamma-butyrolacetone, lycoramine, etc.

### 2.2. Quantification of Galanthamine in In Vivo Grown Plants of Zephyranthes spp.

**Figure 3:**
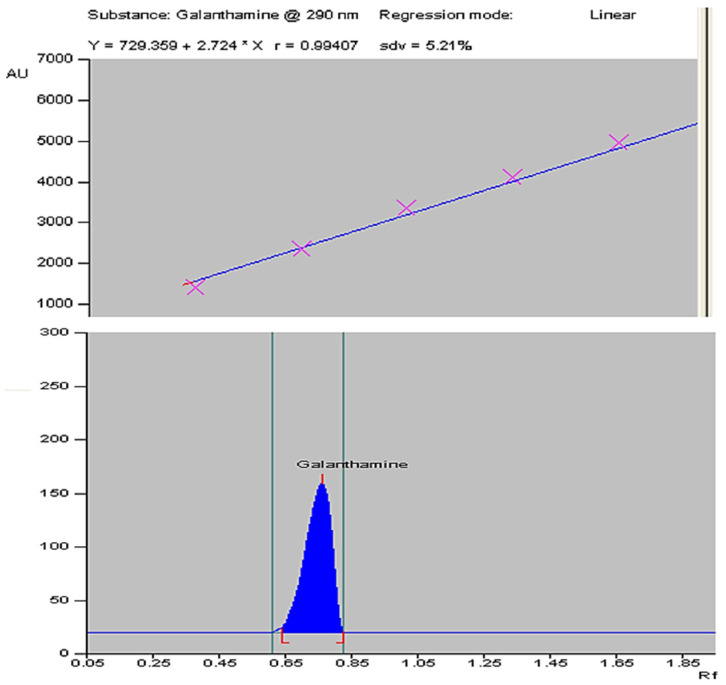
The calibration curve of standard galanthamine with linear regression correlation coefficient r = 0.994 and regression equation y = 729.359 + 2.724 × x, where *y* is the spot area and *x* is the concentration in µg/spot (**top**). HPTLC densitogram displaying single, sharp, and flat peaks of standard Galanthamine at Rf = 0.72, measured at wavelength = 290 nm (**bottom**).

**Figure 4:**
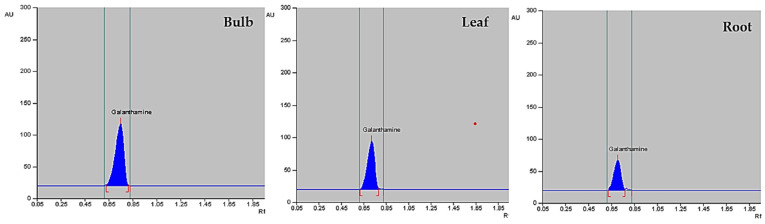
Comparative HPTLC densitograms of bulb, leaf, and root tissues respectively in *Zephyranthes* displaying similar peaks at Rf = 0.72.

The plant material for this study was obtained through in vitro propagation using a previously described method [42]. The galanthamine content was quantified in dried bulb, leaf, and root parts of *Z. grandiflora*, *Z. candida*, and *Z. citrina* by using HPTLC. The six-point calibration curve of galanthamine (Figure 3, top) showed a linear relationship with regression correlation coefficient r = 0.994 and regression equation y = 729.359 + 2.724 × x, where y is the spot area and x is the concentration in µg/spot. The mobile phase chloroform: acetone: ethanol in the ratio of 8:4:1 with saturation time of 1 h displays single sharp, flat and compact peaks at Rf = 0.72, detected on wavelength 290 nm (Figure 3, bottom). The galanthamine peaks of bulb, leaf and root are shown, respectively, in Figure 4.

The content of phytocompound galanthamine from three species of *Zephyranthes* was quantified. The highest amount of galanthamine was present in bulb, followed by leaf and root tissues. The highest content was noted in bulb parts of *Z. candida* (2.41 µg g^−1^ DW), followed by *Z. grandiflora* with 2.13 µg g^−1^ DW and in *Z. citrina* the galanthamine content was 2.02 µg g^−1^ DW. The galanthamine content of the leaf in the *Z. candida*, *Z. grandiflora* and *Z. citrina* was 1.38, 1.23 and 1.12 µg g^−1^ DW, respectively. The galanthamine content in root tissues was 0.61, 0.50, and 0.42 µg g^−1^ DW in *Z. candida*, *Z. grandiflora* and *Z. citrina*, respectively. Thus, the yield of galanthamine in different organs followed this order: bulb > leaf > root, and in species *Z. candida > Z. grandiflora > Z. citrina* (Table 2).

### 2.3. Effect of MJ Dosage on Accumulation of Galanthamine in In Vitro-Derived Plantlets of Zephyranthes spp.

**Figure 5:**
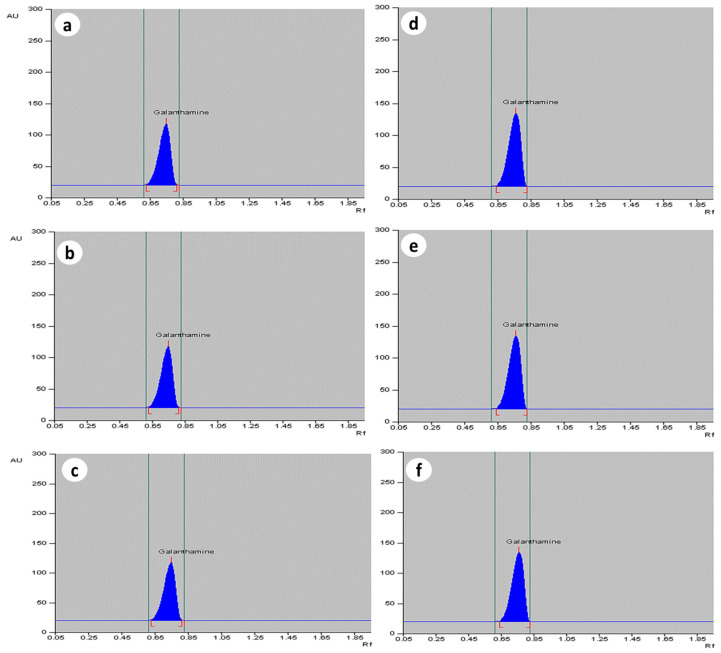
Densitograms of galanthamine content of bulb, leaf, and root tissues on control (**a**, **b** and **c**, respectively), and on elicitation at T3 treatment of methyl jasmonate (**d**, **e** and **f**, respectively).

The MJ elucidated cultures had a higher galanthamine content as compared to the control. The maximum amount of galanthamine was found on T3 treatment of MJ after 2 weeks of treatment, whereas the minimum amount of galanthamine was found on T0 and T4 treatment (Table 3). The highest elicitation effect was observed in bulb (T3 treatment), where galanthamine content increased up to 3.97 µg g^−1^ DW in *Z. candida* when compared to control (2.41 µg g^−1^ DW). On higher than 150 µM dosage of MJ, the galanthamine content decreased. Similarly, in *Z. grandiflora*, the elicitation enhanced galanthamine level, i.e., 2.93 µg g^−1^ DW, as compared to control tissue (2.07 µg g^−1^ DW), and in *Z. citrina*, 2.87 µg g^−1^ DW of galanthamine was noted, as compared to control (2.13 µg g^−1^ DW). In leaf parts, 2.06, 1.97, 1.98 µg g^−1^ DW of galanthamine was accumulated in *Z. candida*, *Z. grandiflora*, and *Z. citrina*, respectively, on T3 dosage of MJ. The roots showed the least elicitation effect and a marginal increase in galanthamine, i.e., 0.72, 0.85, and 0.90 µg g^−1^ DW was noted, as compared to the control. The densitograms on control and T3 treatment for the bulb, leaf, and root extracts are shown in Figure 5 and the yields are shown in Table 3, Table 4 and Table 5 for *Z. candida*, *Z. grandiflora*, and *Z. citrina*.

## 3. Discussion

The potent activity against cancer cells and acetyl cholin esterase activity of Amaryllidaceous alkaloids increased their demand in the pharmaceutical industry. The unavailability of raw plant materials facilitates in vitro culture as an alternative technique for the sustainable production of alkaloids. This method produces uninterrupted, imperishable production of compounds like galanthamine and lycorine. The experiments were conducted here to measure the yield of Amaryllidaceae alkaloids using tissue culture techniques. In this study, shoot induction and multiplication were achieved through a direct regeneration tissue culture system. The bulb-scale explants were placed on MS medium, and the addition of BAP alone or in combination with NAA improved direct shoot induction. The direct shoot regeneration process has the advantage of showing more genetic stability over other methods and was reported in several plant species, such as *Bacopa monnieri* [43], *Jasminum nudiflorum* [44], and *Mansonia altissima* [45], in which BAP, or cytokinin, plays a very important role, as noted in plants like *Musa* sp. [46] and *Aloe vera* [47]. Cytokinins act as signal molecules in regulating plant growth and promote other cellular processes, i.e., rapid cell division, differentiation, apical dome initiation, and the progression of shoots [48,49]. The amendment of BAP and NAA proved an efficient combination in promoting shoot number and growth, as displayed by a number of plants such as *Solanum tuberosum* [50], *Lippia javanica* [51], *Santalum album* [52], and *Quercus robur* [53]. The tissue culture-derived shoots of *Zephyranthes* species were successfully rooted on medium containing IBA. IBA has been reported to induce in vitro rooting in many plant species, such as *Centratherum punctatum* [54], and *Althea officinalis* [55]. IBA has been widely utilized for promoting in vitro rhizogenesis, as noted in *Taxus* [56].

The GC-MS technique has frequently been used for screening and detection of phytocompounds like alkaloids, flavonoids, volatile elements, and other bio-actives [57]. In this study, the chromatographic presentations showed 34 bioactive compounds with variable content in the methanolic extract of *Zephyranthes*. Many of these detected phyto-constituents exhibited protective biological activities against various diseases [58]. Detection and quantification of phytocompounds from in vivo and in vitro-grown medicinal plant species using GC-MS have earlier been observed [59,60]. Here, the quantification of galanthamine from naturally grown plant parts (bulb, leaf, and root) and in vitro grown tissues was made in *Zephyranthes*, and the influence of MJ on yield was measured through high-performance thin layer chromatography (HPTLC). The HPTLC densitograms of standard and galanthamine sample at Rf = 0.72 show a sharp, flat peak, confirming the previously described method [61,62]. The compound was accumulated high in the bulb, followed by the leaf and root in the bulb > leaf > root order. Among the different species evaluated, the highest yield of galanthamine was noted in *Z. candida*, and the maximum galanthamine contents of 2.41 µg g^−1^ DW were detected in bulbs.

Elicitors are grouped into biotic, abiotic, and intracellular signaling elements on the basis of origin and cellular function [63]. The production and accumulation of secondary metabolites depended on elicitor type, concentration, and exposure time [64]. Based on elicitation investigation of in vitro grown tissues, the Amaryllidaceae alkaloid galanthamine showed enhanced yield in tissues. In *Z. candida*, the highest accumulation of galanthamine was noted at 150 µM of MJ, the maximum being 3.97 µg g^−1^ DW as compared to the control (2.41 µg g^−1^ DW). In *Z. grandiflora* and *Z. citrina*, 2.93 and 2.87 µg g^−1^ DW of galanthamine was noted at 150 µM. Among the organs evaluated, the highest accumulation was observed in bulbs, followed by leaves and roots. Thus, MJ proved to be an effective elicitor for enhancing galanthamine. MJ, as an exogenous elicitor, stimulated a cascade of signal transduction pathways in up-regulating stress-related genes/proteins, which in turn enhanced the synthesis of secondary metabolites through extensive cross-talk and transcriptional reprogramming [65]. Here, the elicitation with MJ enhanced galanthamine yield by about 64.73% in in vitro tissues of *Zephyranthes,* and T2/T3 dosages proved to be ideal concentrations in enhancing secondary metabolite yield; similar few-fold enriched synthesis of bioactive compounds was reported in other studied medicinal plants like *Panax ginseng* [66], *Changium smyrnioides* [67], and *Mentha x piperita* [68]. A lower MJ dosage promoted transcripts of genes like ArPAL, ArC4H, and Ar4CL for alkaloid synthesis [69]. The maximum yield of galanthamine in the bulb of Amaryllidaceae may be due to overexpression of biosynthetic regulatory genes like C_4_H (cinnamate-4-hydroxylase) and phenylalanine ammonia-lyase (PAL). A similar observation of the elicitor’s influence on Amaryllidaceae alkaloid yield in bulbs was reported in a recent study [70].

MJ is believed to activate genes involved in producing jasmonic acid, which in turn regulates stress-related proteins through different signal transduction pathways [71]. It is a commonly used elicitor to enhance secondary metabolites and participates in pathways independent of stress hormones like salicylic acid, jasmonic acid, and ethylene [42,72]. The exogenous MJ stress treatments in in vitro-grown tissues trigger a chain of signal transduction, ROS, and antioxidant enzymes like superoxide anion (O_2_^•−^), hydroxyl radical (OH), hydrogen peroxide (H_2_O_2_), singlet oxygen (O_2_) pathways [73]. Thus, the process of elicitation, one of the advanced techniques adopted to enhance secondary metabolites, works on the principle of counteraction of stress [74]. The molecular mode of action of elicitors is not clearly elucidated. Elicitors are known to bind plasma membrane receptors, regulate signal transduction pathways by modulating stress-related genes’ transcription, and enhance the biosynthesis of phytocompounds [75]. This is the first quantitative HPTLC assessment of galanthamine in various plant parts of *Zephyranthes* sp.

## 4. Materials and Methods

### 4.1. Plant Material and Surface Sterilization Method

Three *Zephyranthes* species (*Z. grandiflora*, *Z. candida*, and *Z. citrina*) were collected in the months of March–April from Jamia Hamdard herbal garden, New Delhi, and were certified (Dr. Akhtar H. Malik, Taxonomist, University of Kashmir, India). The outer dry scales were discarded; washing of bulb segments was carried out with commercial detergent cetrimide, which is a mixture of tetradecyltrimethylammonium, dodecyltrimethylammonium, and hexadecyltrimethylammonium. Before inoculation, the bulbs were first surface sterilized with 70% ethanol for one min, later with 0.05% (*w*/*v*) mercuric chloride solution (3 min), followed by three-four-time washing with sterilized distilled water. The bulbs were cut into explants containing condensed stems and scale leaves (also called bulb-scale). The explant was then transferred to test tubes (Borosil, Mumbai, India), containing about 10 mL of MS medium [76]. The surface sterile bulb-scale segments were placed on a medium containing MS salts, 0.8% agar, and 3% sucrose. The medium pH was adjusted to 5.8, and it was autoclaved at 121 °C for 15 min. The test tubes/cultures were kept under white fluorescent light (16 h photoperiod; 55 µmol m^−2^ s^−1^, Philips, Kolkata, India) at 26 ± 2 °C with about 70% relative humidity (RH).

### 4.2. GC-MS Analysis

The methanolic extracts of plants for the GC-MS study were carried out using a GC-MS-QP-2010 (Shimadzu, Tokyo, Japan) model, following an optimized program: helium gas was run at a constant flow of 1.21 mL/min; the incubated temperature 260 °C; the initial oven temperature 100 °C with 3 min holding time, gradually elevated to 300 °C for 17 min. The preferred column used for the separation of compounds was Rxi-5Sil MS GC Capillary, 30 m, 0.25 mm ID, 0.25 µm df. The ion and interface temperatures were kept at 220 °C and 270 °C respectively; the solvent cut time was 2.5 min, and the GC-MS run time for all samples was 35 min. The compounds present in bulb parts were identified by using mass spectral database of the National Institute of Standards and Technology library and comparing the retention indices, peak area, and peak area % with the identified phytocompounds through GC-MS solution software version 4.45 SP1.

### 4.3. Elicitor Preparation and Dosage

The methyl jasmonate, MJ, (Sigma-Aldrich, St. Louis, MO, USA) was used as an elicitor; and the stock solution of MJ was prepared by dissolving a specific amount in 90% ethanol, and the final volume was made with double-distilled water. The in vitro culture/regenerated shoots at their active growth stage (2–3 weeks old) were cultivated on MS with various MJ concentrations i.e., T1 = 50, T2 = 100, T3 = 150, and T4 = 200 µM for fifteen days. The medium without MJ was used as the control (T0).

### 4.4. Extraction Procedure for High Performance Thin Layer Chromatography (HPTLC)

#### 4.4.1. Stock Solution and Sample Extraction Procedure

One (1.0) mg of galanthamine procured from Sigma-Aldrich (St. Louis, MO, USA) was dissolved in 1.0 mL of methanol, making a 1.0 mg mL^−1^ stock solution. A number of concentrations, i.e., 0.3, 0.6, 0.9, 1.2, 1.5, and 1.8 µL, were used on 10 × 10 cm TLC silica plates for standard plot preparation. The various plant parts, like bulbs, leaves, and roots, of natural-grown and in vitro-derived plants from the control and MJ treatments were harvested and shade-dried at 28 ± 2 °C. The dried plant material was ground into powder, and the extraction was made by macerating the powdered sample (100 mg) in 1.0 mL of solvent (methanol:water) (Chempur, Piekary Slaskie, Poland) in 9:1 ratio. Centrifugation of homogenate was made at 13,000 rpm for 25 min to remove impurities. The extract was concentrated at 40 °C by using a rotary evaporator; the residue was dissolved in solvent and filtered through a 0.45 µm syringe membrane.

#### 4.4.2. HPTLC Instrumentation and Chromatographic Conditions

HPTLC was conducted on aluminum plates (20 × 10 cm) coated with 0.2 µm silica gel (Merck SA, Darmstadt, Germany). The aluminum plates were methanol washed to remove impurities and oven dried at 100 °C for 5–10 min. The different levels of sample and standard were spread at a rate of 80 mL/s by a constant flow of N_2_ with a 5 mm band width on the sample applicator Linomat V (CAMAG, Muttenz, Switzerland), equipped with a 100 µL syringe. After sample use, the plates were air dried at room temperature and prepared in a CAMAG twin-through glass chamber, saturated with 1 h mobile phase with linear ascending mode up to 90 mm. The mobile phase was a mixture of chloroform: acetone: ethanol (Sigma-Aldrich, St. Louis, MO, USA) at a ratio of 8:4:1. The plates were scanned at 290 nm wavelength with a TLC scanner V (CAMAG, Muttenz, Switzerland) at a slit dimension of 6.0 × 0.1 mm and a scanning speed of 20 mm/s. The peak areas of the samples were used for the quantification of galanthamine, using the standard peak as a reference.

### 4.5. Statistical Analysis

The influence of PGRs on shoot and root formation and the impact of MJ treatments on galanthamine yield in *Z. grandiflora*, *Z. candida*, and *Z. citrina* were analyzed. The bars in tables and figures indicate the mean ± standard error of three replicates, which were conducted at least twice. The mean values were separated using Duncan’s Multiple Range Test (DMRT) at *p* ≤ 0.05.

## 5. Conclusions

Galanthamine content was determined in three *Zephyranthes* sp. *Z. candida*, *Z. grandiflora*, and *Z. citrina*, and the influence of methyl jasmonate on galanthamine yield was assessed in in vitro grown plant parts. The content was highest in *Z. candida* bulb (2.41 µg g^−1^ dry wt.); the next best source was *Z. grandiflora* (2.13 µg g^−1^ dry wt.) Elicitation with 150 μmol/L MJ resulted in the highest galanthamine content (3.97 µg g^−1^ dry wt.) in *Z. candida* bulb, with an increase of 64.73% compared to the control. The process of elicitation using MJ or other elicitors and their optimized doses could be a useful strategy for enriching the synthesis of important alkaloids of commercial interest.

## Figures and Tables

**Table 1:** Common compounds detected in methanolic extracts of *Zephyranthes bulbs* using GC-MS analysis.

Peak	R.Time	Area	Area%	Name
1	4.173	529325	0.57	N-Isopentyl-N-nitroso-pentylamine
2	4.535	212439	0.23	Methylolacetone
3	4.707	92871	0.10	N-acetyl-LAsparticacid
4	4.801	885370	0.96	2,4-Dihydroxy-2,5-dimethyl-3(2H)-furan-3-one
5	5.250	1473326	1.59	2-Hydroxy-gamma-butyrolactone
6	5.721	5266629	5.70	1,2,3-propanetriol
7	5.964	197792	0.21	Acetic acid, pentylester
8	6.059	263065	0.28	4-Acetylbutyricacid
9	6.174	1048162	1.13	3,4-dihydroxy-3-methylbutyl Acetate
10	6.375	123480	0.13	1,3-dioxolane-4-methanol,2,2- Dimethyl-
11	6.582	5097432	5.51	Cyclopropylmethanol
12	7.038	415911	0.45	5-amino-6-nitroso-2,4(1h,3h)- Pyrimidinedione
13	7.990	7141625	7.72	4H-Pyran-4-one,2,3-dihydro-3,5-dihydroxy-6- methyl-
14	8.141	867823	0.94	2(3H)-Furanone,dihydro-4-hydroxy-3-methylene-
15	8.816	237368	0.26	Carbamicacid,(3,4,4-trimethyl-1,2-dioxetan-3- yl)methyl
16	8.998	935988	1.01	5-Hydroxymethylfurfural
17	9.745	2562191	2.77	1,2,3-propanetriol,diacetate
18	10.365	296036	0.32	2-Methoxy-4-vinylphenol
19	12.421	511122	0.55	.alpha.-D-Galactopyranoside,methyl
20	13.764	43505913	47.05	guanosine
21	15.176	9639265	10.42	alpha.-D-Galactopyranoside
22	17.198	5062691	5.48	Pretazettinealpha.-D-Galactopyranoside,methyl
23	15.176	165523	0.18	Stevioside
24	17.198	1307423	1.41	n-Hexadecanoicacid
25	18.182	311511	0.34	13-Hexyloxacyclotridec-10-en-2-one
26	18.890	1369541	1.48	9-Octadecenoicacid
27	21.636	789504	0.85	Lycoramine
28	22.108	100646	0.11	Lycorenan-7-one,2,4-didehydro-2- Deshydroxy-phenanthridin-1-ol
29	22.526	381154	0.41	4-[1-(1-hydroxy-ethyl)-1h-indol-4-yl]- 2-meth
30	22.703	336879	0.36	Dimethyl2,6-dimethyl-4-(2- Nitrophenyl)-1,4-
31	24.500	78521	0.08	1,3-cyclohexanedicarboxamide, Trans-
32	24.783	312808	0.34	Tazettine
33 34	25.662 29.810	251686 693091	0.27 075	Galanthan-1-ol,9-methoxy-4-methyl-11-oxa-4-azatetracyclotetraen-14-ol Isopropyllinoleate

**Table 2:** The galanthamine content (µg g^−1^ DW) of the bulb, leaf, and root organs in the field grown species of *Zephyranthes*.

Parts Used	*Z. candida*	*Z. grandiflora*	*Z. citrina*
Bulb	2.41 ± 0.04a	2.13 ± 0.04a	2.02 ± 0.03a
Leaf	1.38 ± 0.02b	1.23 ± 0.03b	1.12 ± 0.01b
Root	0.61 ± 0.01c	0.50 ± 0.01c	0.42 ± 0.01c

Values are mean ± standard error of three experiments. Mean values are followed by different letters are significantly different at *p* ≤ 0.05 according to DMRT.

**Table 3:** Accumulation of galanthamine content (µg g^−1^ DW) in bulb, leaf, and roots parts of in vitro derived *Zephyranthes candida* on MS medium containing Methyl Jasmonate.

Parts Used	T0	T1	T2	T3	T4
Bulb	2.41 ± 0.03c	2.51 ± 0.04b	2.58 ± 0.04d	3.97 ± 0.04a	2.02 ± 0.02e
Leaf	1.38 ± 0.04d	1.53 ± 0.03b	1.87 ± 0.02c	2.06 ±0.03a	1.27 ± 0.02d
Root	0.51 ± 0.01c	0.53 ± 0.01b	0.67 ± 0.01d	0.70 ±0.01a	0.54 ± 0.01c

Values are mean ± standard error of three experiments. Mean values followed by different letters are significantly different *p* ≤ 0.05 according to DMRT. T0 = Control, T1 = 50 µM, T2 = 100 µM, T3 = 150 µM, and T4 = 200 µM of MJ.

**Table 4:** Accumulation of galanthamine content (µg g^−1^ DW) in bulb, leaf, and roots parts in *Zephyranthes grandiflora* in treatments with Methyl jasmonate.

Parts Used	T0	T1	T2	T3	T4
Bulb	2.07 ± 0.04c	2.26 ± 0.05b	2.62 ± 0.04c	2.93 ± 0.06a	1.64 ± 0.05d
Leaf	1.43 ± 0.02c	1.52 ± 0.03b	1.74 ± 0.02d	1.97 ± 0.04a	1.01 ± 0.03e
Root	0.60 ± 0.03b	0.61 ± 0.01a	0.63 ± 0.01b	0.65 ± 0.01a	0.47 ± 0.001b

Values are mean ± standard error of three experiments. Mean values followed by different letters are significantly different at *p* ≤ 0.05 according to DMRT. T0 = Control, T1 = 50 µM, T2 = 100 µM, T3 = 150 µM, and T4 = 200 µM of MJ.

**Table 5:** Accumulation of galanthamine content (µg g^−1^ DW) in bulb, leaf, and roots parts of *Zephyranthes citrina* on different treatment of Methyl jasmonate.

Parts Used	T0	T1	T2	T3	T4
Bulb	2.13 ± 0.03c	2.45 ± 0.04c	2.66 ± 0.05b	2.87 ± 0.02a	2.06 ± 0.04b
Leaf	1.22 ± 0.01c	1.43 ± 0.02d	1.65 ± 0.03b	1.98 ± 0.003a	1.32 ± 0.01d
Root	0.34 ± 0.01c	0.37 ± 0.01d	0.40 ± 0.01b	0.42 ± 0.001a	0.12 ± 0.006e

Values are mean ± standard error of three experiments. Mean values, followed by different letters are significantly different at *p* ≤ 0.05 according to DMRT. T0 = Control, T1 = 50 µM, T2 = 100 µM, T3 = 150 µM, and T4 = 200 µM of MJ.

## Data Availability

All data are available in the article.
